# Molecular Genetic Characterization of Individual Cancer Cells Isolated via Single-Cell Printing

**DOI:** 10.1371/journal.pone.0163455

**Published:** 2016-09-22

**Authors:** Julian Riba, Nathalie Renz, Christoph Niemöller, Sabine Bleul, Dietmar Pfeifer, Juliane M. Stosch, Klaus H. Metzeler, Björn Hackanson, Michael Lübbert, Justus Duyster, Peter Koltay, Roland Zengerle, Rainer Claus, Stefan Zimmermann, Heiko Becker

**Affiliations:** 1 Laboratory for MEMS Applications, Department of Microsystems Engineering - IMTEK, University of Freiburg, Freiburg, Germany; 2 Department of Medicine I, Medical Center - University of Freiburg, Freiburg, Germany; 3 Faculty of Medicine, University of Freiburg, Freiburg, Germany; 4 Department of Internal Medicine III, University of Munich, Munich, Germany; 5 Hahn-Schickard Society for Applied Research, Freiburg, Germany; 6 BIOSS – Centre for Biological Signalling Studies, University of Freiburg, Freiburg, Germany; University of Navarra, SPAIN

## Abstract

Intratumoral genetic heterogeneity may impact disease outcome. Gold standard for dissecting clonal heterogeneity are single-cell analyses. Here, we present an efficient workflow based on an advanced Single-Cell Printer (SCP) device for the study of gene variants in single cancer cells. To allow for precise cell deposition into microwells the SCP was equipped with an automatic dispenser offset compensation, and the 384-microwell plates were electrostatically neutralized. The ejection efficiency was 99.7% for fluorescent beads (n = 2304) and 98.7% for human cells (U-2 OS or Kasumi-1 cancer cell line, acute myeloid leukemia [AML] patient; n = 150). Per fluorescence microscopy, 98.8% of beads were correctly delivered into the wells. A subset of single cells (n = 81) was subjected to whole genome amplification (WGA), which was successful in all cells. On empty droplets, a PCR on *LINE1* retrotransposons yielded no product after WGA, verifying the absence of free-floating DNA in SCP-generated droplets. Representative gene variants identified in bulk specimens were sequenced in single-cell WGA DNA. In U-2 OS, 22 of 25 cells yielded results for both an *SLC34A2* and *TET2* mutation site, including cells harboring the *SLC34A2* but not the *TET2* mutation. In one cell, the *TET2* mutation analysis was inconclusive due to allelic dropout, as assessed via polymorphisms located close to the mutation. Of Kasumi-1, 23 of 33 cells with data on both the *KIT* and *TP53* mutation site harbored both mutations. In the AML patient, 21 of 23 cells were informative for a *TP53* polymorphism; the identified alleles matched the loss of chromosome arm 17p. The advanced SCP allows efficient, precise and gentle isolation of individual cells for subsequent WGA and routine PCR/sequencing-based analyses of gene variants. This makes single-cell information readily accessible to a wide range of applications and can provide insights into clonal heterogeneity that were indeterminable solely by analyses of bulk specimens.

## Introduction

Intratumoral clonal heterogeneity may impact treatment response to chemotherapy or targeted therapies and hence the outcome of cancer patients [1,2]. Information on gene mutations derived from next generation sequencing (NGS) of bulk cell populations has been increasingly used to gain insights into the clonal heterogeneity of malignancies. However, this bioinformatically inferred data may only give an approximation of the definite clonal architecture. Single-cell genotyping is necessary to verify the co-existence of mutations in a cell and to derive reliable information about the clonal architecture and evolution of a disease.

Genetic information on the single-cell level has become more accessible in the recent years. This led to several studies which revealed deeper insights into the clonal architecture and evolution of various types of solid cancers and leukemias, all of which highlighted the importance of single-cell analyses [3–10]. As we and others have shown for acute myeloid leukemia (AML), single-cell sequencing is particularly useful for verifying the clonal architecture concluded from NGS data and for resolving the clonal assignment of mutations when NGS provides ambiguous or complex clonal architectures [6–9].

Prerequisites for accurate single-cell analyses are the efficient isolation of cells from the bulk sample and their precise deposition into reaction vessels for downstream analyses. Various methods for single-cell isolation have been developed which are more or less suitable depending on the downstream application [11,12]. Among the most frequently used approaches is fluorescence-activated cell sorting (FACS) which allows for high throughput isolation of single cells [13]. However, FACS does not provide a direct proof that truly a single cell was isolated; moreover, the integrity of the cells may be compromised by the shear forces inherent to the system. More recently, various microfluidic approaches have been introduced such as hydrodynamic cell trapping as utilized by Fluidigm´s C1 system [14]. However, these are limited in their flexibility of applications due to a determined chip design. In addition to such automated methods, single cells can be also picked manually with high precision by a microscope-assisted device but only at limited numbers.

The Single-Cell Printer (SCP), that we developed and that was used in the present study, is capable of isolating and depositing single cells with high viability rates in a label-free and non-contact manner [15] and has been previously used for single-cell PCR on human B-cells [16]. Here, we further improve the droplet placement of the SCP to facilitate precise cell deposition into the center of the wells of standard 384-microwell plates. Furthermore, we study gene mutations and polymorphisms in cancer cells using routine PCR and Sanger sequencing after whole genome amplification (WGA) in order to evaluate the co-occurrence of mutations in individual cells and the clonal genetic architecture.

## Materials and Methods

### Cell lines and patient sample

The osteosarcoma-derived cell line U-2 OS (LINTERNA^™^ U-2 OS) was received from Innoprot (No. P20116; Derio, Spain) and cultured in DMEM/F12-Ham medium plus 10% FBS, 1% penicillin/streptomycin and 10 μg/ml puromycin in a humidified 5% CO_2_ atmosphere; for harvesting, cells were trypsinized. The AML-derived cell line Kasumi-1 was received from the research group of Michael Lübbert (University of Freiburg) who obtained it from DSMZ (No. ACC 220; Braunschweig, Germany); the cells were cultured in RPMI medium plus 10% FBS and 1% penicillin/streptomycin in a humidified 5% CO_2_ atmosphere. Furthermore, peripheral blood mononuclear cells (PBMCs) of a patient with AML were used (metaphase karyotype: 90–91, XXYY, -2, -5, +13x2, add(17)(p11), -21x2[cp8], 46,XY[3]; interphase fluorescence in situ hybridization (FISH): 90% of cells had 3 signals for 17p13). Written informed consent was obtained from the patient prior to sampling. Sampling and research were approved by the ethics committee of the University Freiburg on 13. June 2013 and under the reference no. 464/11. At the time of sampling, the peripheral blood of the patient contained 82% blasts as assessed by cytomorphology. The blood sample was enriched for mononuclear cells through Ficoll-Hypaque and cryopreserved until use. Genomic DNA was extracted from the bulk samples using the AllPrep DNA/RNA Kit (Qiagen).

### Sample preparation and single-cell printing

In principle, the SCP was used as described before [15]. Prior to cell printing in the current study, the cells were re-suspended in PBS to yield a final concentration between 10^5^ and 10^6^ cells/ml. For each experiment, a new sterile cartridge with a 40 μm nozzle was filled with 30 μl sample and mounted on the SCP. The piezo stroke length was set to 10 μm and the downstroke velocity was set to 140 +/- 10 μm/s to achieve stable droplets. Individual cells were printed into the wells of a standard 384-microwell PCR plate. Electrostatic charges on the plates were neutralized with an ionizing air blower (minION2, SIMCO-ION, The Netherlands). Sample loading and instrument preparation took on average approximately 5 minutes.

### Calculation of the dispenser offset

In order to compensate for dispenser offset, reference droplets were dispensed on a hydrophobic-coated glass slide that is imaged by a digital camera (DFM 72BUC02-ML, The Imaging Source, Germany) from below. The position of the droplet was then calculated from the image data by the SCP software using the.NET openCV wrapper Emgu CV. This fully automated process was carried out prior to the cell deposition and each time the offset was taken from the mean of three subsequently dispensed droplets. After completion of the single-cell deposition the dispenser offset was assessed again for comparison with the offset measured at the start of the experiment. By this means, a drift in the dispenser offset that could potentially occur during the experiment can be automatically detected. However, in this study, we experienced no significant drift that would compromise the precision of cell deposition into the microwells.

### Metrics for the single-cell isolation performance

Single-cell isolation performance of the SCP was characterized using two measures. The ejection efficiency is defined as the number of single beads or cells ejected from the nozzle divided by the total number of printing events targeted to the microwell plate. The ejection efficiency is determined from the SCP images and is therefore independent of the target (e.g well). The deposition efficiency quantifies the number of single beads or cells correctly delivered to the bottom of the microwells. Since single fluorescent cells cannot always be clearly visualized on the microwell bottoms due to auto-fluorescence caused by the rough well surface, we used high-intensity fluorescent beads (Kisker Biotech, Germany) to determine the deposition efficiency.

### Detection of nucleotide variants in the bulk specimens

Candidate gene mutations in the cell lines were selected via the COSMIC mutation database [17] and confirmed by Sanger sequencing (U-2 OS, Kasumi-1) or MiSeq-based targeted NGS (Kasumi-1) of bulk specimens. In the patient sample, variants were assessed by Sanger sequencing. Variant allele frequencies (VAFs) in Kasumi-1 were derived from NGS or pyrosequencing.

In the targeted NGS assay, 70 genes reported to be mutated in AML or other hematologic cancers were analyzed by multiplexed amplicon resequencing (Agilent HaloPlex); sequencing was performed on an Illumina MiSeq platform using 2 x 250 bp paired-end reads [18]. Pyrosequencing was performed according to the manufacturer’s instruction using a PyroMark Q96 MD system.

CytoScan HD or Human SNP Array 6.0 arrays (Affymetrix) were performed on the bulk samples. The data were analyzed using the Chromosome Analysis Suite (ChAS) software. For the evaluation of copy number variations (CNVs), the normalized probe intensities for the A and B alleles were summarized to calculate allelic signal values. The allelic difference was calculated as the difference between the signal of the A allele minus B allele, and standardized so that an A allele genotype has a positive value and a B allele a negative value. The standardization was based on median values for the allelic difference under different genotype configurations determined by the reference set. The array data are deposited at http://www.ebi.ac.uk/arrayexpress/ (Acc. E-MTAB-4950). In addition, publically available SNP Array 6.0 data (NCBI GEO database acc. GSM879223) were used to identify heterozygous single nucleotide polymorphisms (SNPs) in U-2 OS (see below).

### Whole-genome amplification of single-cell DNA

The printed cells were lysed and the DNA of each cell was amplified by WGA using the REPLI-g Single Cell Kit (Qiagen), which is based on multiple displacement amplification. The manufacturer’s protocol was modified in terms of a four-fold reduction of all reagents resulting in a final reaction volume of 12.5 μl. The DNA yield was assessed by Qubit fluorometric quantitation (Thermo Fisher Scientific) according to the manufacturer’s protocol. In addition, a multiplex PCR on repetitive *LINE1* retrotransposons was used to evaluate the DNA after the WGA. This PCR generates specific products of different sizes irrespective of whether specific genomic regions were amplified better or worse by the WGA, and are thus over- or underrepresented. The *LINE1* primers are provided in S1 Table.

### Single-cell genotyping

For single-cell genotyping, the WGA DNA from the individual cells was subjected to PCR and Sanger sequencing of the selected variant loci. As WGA can lead to the preferential amplification of one allele and allelic dropout (ADO), we also sequenced SNPs that were identified by CNV array to be heterozygous in the bulk sample and located in close genomic proximity to the respective mutation loci. If, in a single cell, there was no mutated sequence at the mutation site and the respective SNP not heterozygous, then ADO may have occurred at the genomic locus, and the mutation analysis was deemed to be inconclusive. The primers are provided in S1 Table.

## Results

### The SCP and its implementation into the single-cell genotyping workflow

**Fig 1:**
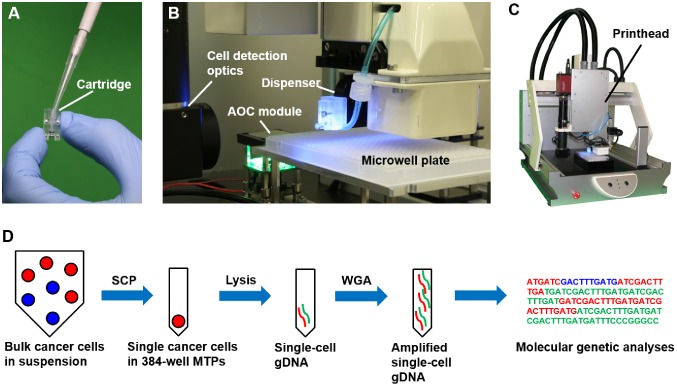
Single-cell genotyping workflow implementing the Single-Cell Printer. **(A)** The cell suspension is filled into the sterile single-use cartridge. **(B)** The microwell plate holder is equipped with a camera to automatically determine and adjust for the dispenser offset prior to cell printing (automatic offset compensation, AOC). The dispenser with the mounted cartridge and the cell detection optics are part of the printhead. **(C)** Total view of the SCP prototype that was used in this study. **(D)** Illustration of the workflow for single-cell genotyping. Individual cells are isolated via the SCP. After cell lysis, the DNA is subjected to whole genome amplification (WGA), which then can be used for routine molecular genetic analyses.

The original SCP principle has been previously described in detail [15]. Fig 1A–1C show the SCP prototype that we used for the present study and the workflow for single-cell isolation and analysis in the 384-well format: First, the cell suspension is pipetted into the disposable cartridge that consists of a milled plastic part and the microfluidic dispenser chip (Fig 1A). Next, the cartridge is mounted on the printhead that comprises the piezo actuator driving the dispenser chip (Fig 1B). A microscopic vision system monitors the nozzle of the dispenser chip and provides the image data for cell detection, classification and isolation (see below). Unwanted droplets are discarded via a vacuum shutter system. The printhead is mounted on a three-axis robotic stage which allows precise deposition of single-cell encapsulating droplets into microwells specified in the SCP software by the operator. Different from previous applications, in the present study, the SCP was used for the isolation of cancer cells and subsequent cell lysis, WGA and molecular genetic analyses (Fig 1D).

### Evaluation of the precision and efficiency of single-cell deposition

A prerequisite for the genetic analyses is the exact deposition of the single cell in the well, since only then the cell lysis and WGA can be reliably performed, given the small reaction volumes.

**Fig 2:**
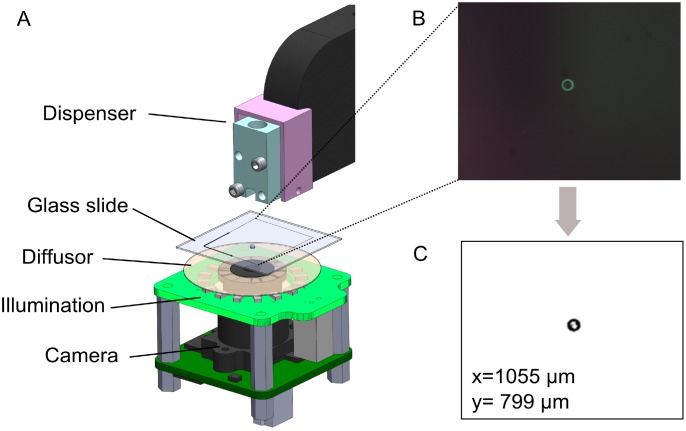
Automatic dispenser offset compensation (AOC) by measuring droplet placement position before / during the cell isolation process. **(A)** For this, droplets are dispensed on a glass slide that is imaged by a digital camera. **(B)** The actual droplet position is extracted from the image data by image processing with openCV. **(C)** displays the binary image after thresholding. The algorithm automatically calculates the correct dispensing position to target the center of the microwell.

Although the precision of the dispenser is sufficiently high to deposit single-cell encapsulating droplets into microwells, we observed that the droplet position within the well can vary. The reason is a variation of the nozzle position due to the cartridge fabrication process and the fixation of the cartridge to the printhead. In order to deposit droplets accurately onto the well bottom in an automated manner we designed a tool to compensate for such an offset by measuring the droplet placement position before and during the cell isolation process. For this, droplets are dispensed on a glass slide that is imaged by a digital camera attached to the microwell plateholder (Fig 2). The droplet position is extracted from the image data and the algorithm automatically calculates the correct dispensing position to target the center of the microwells.

In addition, free-flying droplets can be deflected by electrostatic forces, which occur due to the electric charge that accumulates on both the droplet and plate. Thus, we used ionized air to neutralize the electrostatic charging of the microwell plate.

**Fig 3:**
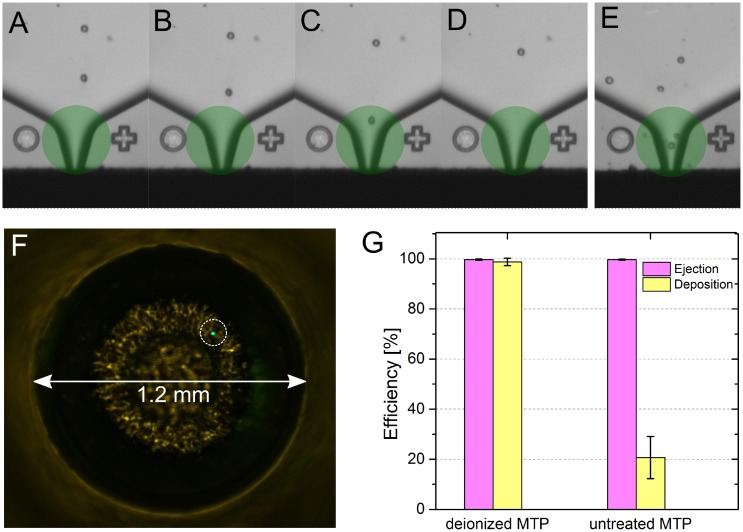
Efficiency of single-cell ejection and deposition into microwells. Four consecutive images are stored automatically for each printing event: **(A-C)** A cell (or bead as cell equivalent) is transported towards the nozzle of the dispenser-chip, where it is detected and classified within a region of interest (ROI, green area). Only if the object recognition meets predefined criteria in terms of size, roundness and singularity, the droplet ejected from the nozzle will be targeted to the well. **(D)** A final image confirms the absence of the cell in the nozzle after droplet ejection. The image series can be used to provide direct evidence that truly a single cell was ejected. **(E)** shows an example for an image where two cells would enter the droplet. Such droplets are automatically discarded by the vacuum suction. To evaluate the precision of the instrument, 2304 single fluorescent beads were printed into six 384-microwell plates. The images were evaluated to determine the ejection efficiency (99.7%). **(F)** Correctly deposited beads (dashed circle) were visualized by fluorescence microscopy of the well bottoms (1.2 mm in diameter). **(G)** The beads were correctly delivered in an average of 98.8% of the wells if the microwell plate was electrostatically neutralized before printing.

In order to evaluate whether the SCP with the automatic dispense offset compensation and the deionization deposits single droplets with high efficiency and precision, single 10 μm sized green fluorescent latex beads as cell equivalents were printed into the wells of a 384-microwell plate, and the ejection and deposition efficiencies were assessed. The ejection efficiency (i.e. truly a single bead has been ejected from the nozzle) was determined through the images automatically stored by the SCP that show the nozzle before, during, and after the dispensation (Fig 3A–3E). The deposition efficiency (i.e. a single bead was successfully delivered to the bottom of the well) was concluded from fluorescence microscopy images (Fig 3F).

Of the fluorescent beads, 1152 each were dispensed into three untreated or three deionized 384-well plates; this equaled a total number of 2304 beads. The overall single-bead ejection efficiency was on average 99.7 ± 0.3%. The deposition efficiency depended on whether the plate was deionized or not. Without prior deionization, in only 20.7 ± 8.4% of the well bottoms a single bead was detected, while after deionization 98.8 ± 1.5% of the beads were correctly delivered (Fig 3G).

Following this workflow, a total of 150 single cells of different origins (U-2 OS, n = 40; Kasumi-1, n = 44; AML patient, n = 66) were printed into deionized 384-well plates resulting in a total single-cell ejection efficiency of 98.7%. A subset of the printed cells of each specimen was subjected to WGA and genotyping (as detailed below).

### Whole genome amplification of single-cells

Single cells were subjected to WGA prior to downstream molecular analysis. In order to minimize the odds for contaminating DNA that would be co-amplified by the WGA, we worked with DNA-free cartridges and plates and reduced the hands-on steps during cell isolation, lysis and amplification. The success of the WGA was assessed by fluorometric quantitation of the DNA, and a PCR on repetitive *LINE1* transposons was used to control for the amplification of human DNA in the samples and its absence in the no-template control.

**Fig 4:**
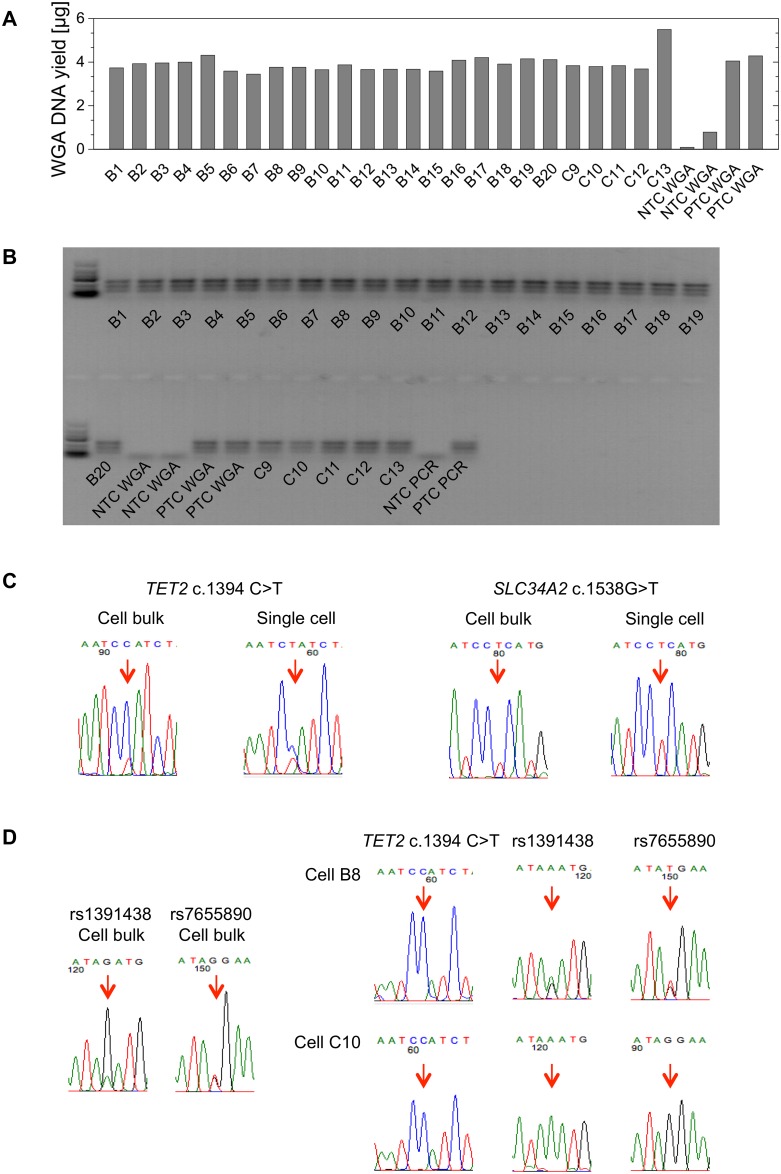
Single-cell whole genome amplification and sequencing of the U-2 OS cell line. **(A)** Bar diagram displaying the WGA DNA yields from the individual U-2 OS cells and the respective controls, as measured by Qubit^™^. **(B)** Agarose gel illustrating the differently sized products of the *LINE1* multiplex PCR that was performed on the WGA DNA of the individual U-2 OS cells. **(C)** Exemplary sequencing chromatograms of the *SLC34A2* and *TET2* gene mutations in the cell bulk and individual cells. **(D)** Conclusions on the occurrence of allelic dropout (ADO) through sequencing of single nucleotide polymorphisms (SNPs). SNPs rs1391438 and rs7655890 are located in close genomic proximity to the *TET2* mutation and show heterozygous patterns in the cell bulk (left). In the single U-2 OS cells B8 and C10, wild-type only is detected at the *TET2* mutation site. The heterozygous patterns of the SNPs in B8 suggest true wild-type in *TET2*, while the detection of only one allele of both SNPs in C10 suggest loss of the genomic region due to ADO. NTC: no-template control, PTC: positive control.

Of the 40 U-2 OS cells that were deposited into the deionized microwells, 25 were subjected to a WGA resulting in a median DNA yield of 3.8 μg (range, 3.5–5.5 μg) per cell (Fig 4A); the PCR on the *LINE1* transposons was positive in all samples (Fig 4B). Of Kasumi-1, 33 cells were subjected to WGA, resulting in a median DNA yield of 14.3 μg (range, 8.6–20.8 μg) per cell, and the *LINE1* PCR was positive in all cells (S1 Fig). Among the 23 single cells from an AML patient, the WGA resulted in a median DNA yield of 16.3 μg (range, 14.0–19.3 μg) per cell and a positive *LINE1* PCR in all cells (S2 Fig).

We also examined whether free-floating DNA was present in the droplets generated by the SCP. Such DNA, if amplified by the WGA, would hinder the genetic analyses of the single cells. Therefore, empty droplets (n = 1, 3, and 10, respectively) from a suspension of Kasumi-1 cells were printed into individual wells of a 384-well plate and then subjected to WGA. All empty droplets yielded no product in the subsequent *LINE1* PCR, while droplets containing single Kasumi-1 cells were positive (S3 Fig).

### Genotyping of single cancer cells

We sought to evaluate the applicability of the SCP for the isolation and genetic analyses of single cancer cells. For this, we studied representative gene variants in U-2 OS, Kasumi-1 and the PBMCs of a patient with AML.

**Fig 5:**
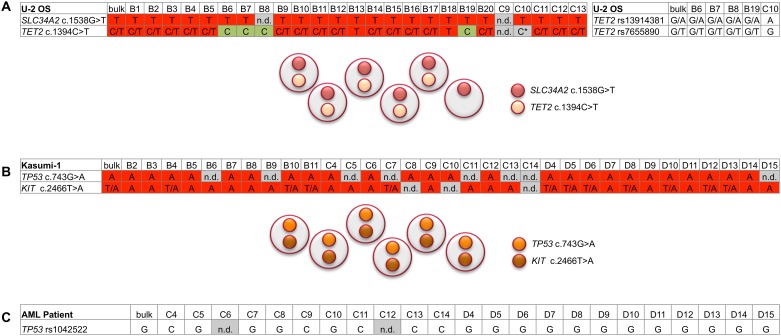
Summary of the genotyping results. **(A)** U-2 OS cell line, **(B)** Kasumi-1 cell line and **(C)** AML patient. Displayed are the nucleotides identified by sequencing of the bulk specimens and the individual cells (annotated for example B1 or C1). Highlighted in red is the presence and in green the absence of the respective mutated sequence. Highlighted in grey are inconclusive analyses either due to failed PCR (n.d., not determined) or the likely occurrence of allelic dropout (*). For the gene mutation analyses, the clonal architecture concluded from the single-cell analyses is schematically displayed.

U-2 OS harbors mutations in the *SLC34A2* (ENST00000382051: c.1538G>T; p.R513L) and *TET2* genes (ENST00000380013: c.1394C>T; p.P465L) [17], both of which are of functional relevance in cancers [19–21]. We confirmed the mutations in *SLC34A2* and *TET2* in the bulk sample. In line with published data [17], the chromatograms suggested that the *SLC34A2* mutation was homo- or hemizygous and the *TET2* mutation heterozygous. From our CNV array data, we concluded that the zygosity of the *SLC34A2* mutation was due to the loss of heterozygosity (LOH) of the respective genomic region. In the 25 U-2 OS cells analyzed, the *SLC34A2* mutation was detected in 23 and the *TET2* mutation in 19 cells (Figs 4C and 5A). In one cell, the *SLC34A2* PCR and, in another cell, both the *SLC34A2* and *TET2* PCR repeatedly failed, which suggests insufficient amplification of the target region by the WGA. As expected from the zygosity in the bulk, no cell with *SLC34A2* wild-type sequence was detected. In contrast, *TET2* wild-type sequence only was detected in 5 cells. These cells were evaluated for the presence of ADO to allow conclusions regarding the co-occurrence of mutations in the individual cells (see below).

Kasumi-1 harbors mutations in the tyrosine kinase *KIT* (ENST00000288135: c.2466T>A; p.N822K) and the tumor suppressor *TP53* (ENST00000269305: c.743G>A; p.R248Q) [17]. The mutations in *KIT* and *TP53* were confirmed by NGS in a Kasumi-1 bulk sample. As verified by pyrosequencing, the VAF of the *KIT* mutation was 84.0% at median (range, 83.3–85.2%). The overrepresentation of the mutated allele is due to the amplification of the *KIT* genomic region [22,23]. The *TP53* mutation was present with a VAF of 100% in line with an LOH of the chromosome 17p region in the CNV array. The *KIT* mutation was detected in 30 and the *TP53* mutation in 25 cells (Fig 5B). In the remaining cells, the *KIT* or *TP53* PCR failed, most likely due to an inefficient amplification of the respective regions by the WGA. No cell with *KIT* or *TP53* wild-type sequence only was detected. With regard to the co-occurrence of the mutations, the analyses yielded informative results for both mutations in 23 cells. All these cells harbored both the *KIT* and *TP53* mutation (Fig 5B).

In the PBMCs of a patient with AML, we assessed the potentially pathogenic non-synonymous SNP rs1042522 in *TP53* (ENST00000269305: c.215C>G; p.P72R) [24,25]. We decided for this approach since no C-allele was detectable by Sanger sequencing of the bulk specimen and since, as indicated by FISH and CNV array, the AML harbored one or more clones with loss of a chromosome 17p allele (including *TP53*; S4 Fig), and chromosomal loss of *TP53* in cancers preferentially affects the C-allele of rs1042522 [25]. Thus, we tested whether an ancestral C-allele would be still detectable in a subset of individual cells. Indeed, the *TP53* PCR yielded a product in 21 cells, in 5 of which the C-allele was detected (Fig 5C).

### Evaluation of allelic dropout and co-occurrence of mutations in U-2 OS

As stated above, in 5 U-2 OS cells the *TET2* wild-type sequence only was detected. To evaluate whether the absence of *TET2* mutations in these cells was due to ADO, we analyzed the SNPs rs1391438 and rs7655890 (located 4,650 bp and 15,992 bp 5’ from the *TET2* mutation, respectively) in these cells; both SNPs were heterozygous in the bulk sample (Fig 4C). In one of the 5 cells, only one of the two alleles of each SNP was detected, which strongly suggests that ADO has occurred at the genomic region that included the SNPs and *TET2* mutation (Fig 4C). Thus, we deemed the analysis of this one cell inconclusive. In the remaining 4 cells, both of the SNPs were heterozygous, suggesting that WGA has successfully amplified both alleles (Fig 4C); this makes it unlikely that the absence of the *TET2* mutation in these cells was due to ADO. Thus, we concluded that these 4 cells indeed lacked the *TET2* mutation.

Thus, in terms of co-occurrence, our analyses yielded informative results for both mutation sites in 22 cells. Of these, 18 cells harbored both the *SLC34A2* and *TET2* mutation while 4 harbored the *SLC34A2* but not the *TET2* mutation, which indicates clonal heterogeneity with regard to *TET2* mutated cells within the U-2 OS cell line (Fig 5A).

In Kasumi-1, no evaluation of ADO was necessary since no cell with *KIT* or *TP53* wild-type sequence only was detected.

## Discussion

Single-cell analyses are the gold standard for deciphering the genetic clonal architecture of solid and blood cancers. Yet, current techniques most often assess gene mutations in the entire population of malignant cells without taking into account that cells might differ genetically from each other. Here, we assessed gene mutations and polymorphisms in bulk samples and individual cancer cells, which were isolated using an improved version of the SCP.

We previously demonstrated that the SCP allows for the isolation and deposition of individual cells with minor impact on cell integrity [15]. The sterile single-use cartridges can be operated with sample volumes as low as 5 μl, have a minimal dead volume, and avoid cross-contamination, as they are the only part that is in contact with the sample. Moreover, the image data that the SCP collects and stores during each experiment, allows for verification that truly a single cell was ejected from the nozzle. Potential double-cell events (which did not occur during this study) can be therefore excluded from downstream analysis without additional imaging of the microwells. The images can be also used for interrogating basic morphological properties such as size and roundness of the printed cell. In summary, the SCP provides the required premises to isolate individual cells from even precious samples for subsequent molecular analyses.

In order to allow accurate cell deposition, for the present study, we improved our established SCP by an automated dispense position measurement system which ensures that the droplets are accurately dispensed onto the specified target positions without any additional user interaction. The system also allows monitoring whether the dispenser delivers stable droplets throughout the cell isolation process. This is a benefit compared to FACS devices, where manual fine-tuning prior to cell isolation is required, including definition of the sorting parameters (fluorescence intensity, forward and side scatter) or alignment of the plate-sorting unit to the microwells.

The single-cell containing droplets that are ejected from the SCP are 160 pl in volume and have velocities of typically 2 m/s. In case of spontaneous electrostatic droplet charging [26], such droplets can be deflected by electrostatic forces due to interaction with the substrate (i.e. the microwell plate). In order to avoid such deflection the droplets or substrate need to be free of electrostatic charge. Here, we successfully applied ionized air to neutralize the charging on the plate. This resulted in droplet trajectories without significant deflection, which was verified by the precise deposition of fluorescent beads onto the bottom of the microwells yielding a deposition efficiency of 98.8%. This is significantly higher than the single-cell trapping efficiencies that are frequently achieved with hydrodynamic cell trapping in microfluidic chips [14].

In the current study, we used the SCP for the isolation of individual cells of cancer cell lines and a patient AML sample to study gene variants that we had detected in the respective bulk specimens. For this, a total of 81 of the printed cells were subjected to WGA to generate sufficient DNA for the genetic analyses. The WGA was successful in all cells, and the DNA yields were uniform and high across the cells isolated per SCP run. In addition, the four-fold reduction of WGA reagents, that we applied in our protocol, implied a profound reduction in costs.

The single-cell containing droplets that are ejected from the SCP are very small, as mentioned above; they are approximately 20x less in volume than the droplets typically produced by FACS. This minimizes the possibility of free-floating DNA in the medium that surrounds the single cell. We nevertheless evaluated the presence of free-floating human DNA in the droplets by printing empty droplets (i.e. not containing a cell, as verified by the SCP image data) from a cell suspension and subjecting these droplets to WGA. While the *LINE1* PCR yielded products for the cell-containing droplets, it remained negative for all empty droplets. This indicates that free-floating human DNA in the droplets is, if at all, a rare event, which clearly improves the reliability of the single-cell data gained from SCP experiments.

An issue of WGA on single-cell DNA is the potential occurrence of ADO. This is of minor importance if the wild-type allele is not amplified, since the mutation will still be detectable. However, if the mutated allele is affected, then this can lead to false interpretation of a cell as being wild-type in the respective gene. In order to control for ADO of the mutated allele, we analyzed cases with the detection of wild-type only for SNPs that were heterozygous in the bulk specimen and located in genomic proximity to the mutation site. If the SNPs were not heterozygous (i.e. lost one allele) in the single cell, then the mutated allele was deemed to be lost by ADO. In U-2 OS, *TET2* wild-type only was identified in 5 cells. Based on the SNP analyses we concluded that 4 of the 5 cells indeed did not harbor the *TET2* mutation (as both alleles of the SNPs were detected), while in one cell the *TET2* mutated allele may have been lost due to ADO (as only one allele of the SNPs was detected). This implies that SNP and mutation sites are present in the same WGA fragments. This is the more probable the closer the sites are located to each other; ideally, they are covered by same PCR amplicon, which is rarely possible. However, WGA fragments have an average product length of more than 10kb [9, 27]. Thus, despite the residual uncertainty, the SNPs that we used were likely suitable to control for ADO of the *TET2* mutation site.

The analyses of the overall 81 single cells yielded 133 successful sequencing reactions and allowed insights into the clonal genetic architecture of the samples.

Among the 22 U-2 OS cells with results for both the *SLC34A2* and *TET2* mutation, 18 cells harbored both mutations and 4 harbored the *SLC34A2* but not the *TET2* mutation. This indicates that U-2 OS consists of clones that are heterogeneous with regard to the *TET2* mutation status. Our findings are in line with the absence of *SLC34A2* wild-type but presence of both *TET2* wild-type and mutated sequences in the bulk sample.

In the 33 Kasumi-1 cells, the *TP53* PCR failed in 8 cells, likely since the respective region on chromosome 17p did not amplify during the WGA, despite the overall success of the WGA in these cells. The amplification failure of the *TP53* region may be attributable to the LOH of the 17p arm in Kasumi-1, since in this case only one allelic copy (as opposed to two in a diploid cell) serves as template for the WGA. Of the 23 cells with information on both *KIT* and *TP53*, all cells harbored both mutations.

Among the 23 single cells of the AML patient, the analysis of the *TP53* polymorphism was successful in 21 cells. In the bulk sample, the cytogenetic data indicated the loss of one copy of the *TP53* encoding chromosomal region, and no C-allele was detectable by Sanger sequencing. Based on this and considering the poor sensitivity of Sanger sequencing, the ancestral genotype in the *TP53* SNP may have been G/G or G/C. From the analyses we concluded that the ancestral genotype was G/C and that the C-allele was affected by the chromosomal deletion. This is in line with the observation that chromosomal loss of *TP53* preferentially affects the C-allele [25,28].

In addition to previous reports by us and others [6–9] we here display examples on how information at the single-cell level can complement and extend the genetic information derived from bulk specimens. The single-cell genotyping allowed verifying the co-occurrence of variants in a given cell, which is essential since only variants that co-exist in a cell can indeed impact together on the biology of this cell. Moreover, the verification or falsification of the co-occurrence of variants allowed conclusions regarding the clonal architecture and evolution.

We are aware that our study has limitations. The number of cancer samples, cells per sample and mutations per cell that we analyzed is relatively small; the analyses of more cells and mutations would allow a more profound insight into the possibilities and limitations of our technique as well as into the clonal architecture of the samples.

However, our study for the first time demonstrates how the SCP, that we had previously established and the precision of which we have further improved for the present study, is used to isolate individual cancer cells in a highly automated manner for molecular genetic analyses. Of particular significance is that, on the cells isolated with the SCP, we obtained uniform and high DNA amounts through WGA and applied routine downstream gene analyses. These features of the SCP and the prior identification of candidate variants in bulk specimens, which then can be studied for co-occurrence in relatively few cells, also demonstrate how meaningful results are obtained from single-cell genotyping at relatively low costs. The costs are indeed further lowered as one SCP cartridge can be used for the isolation of any number of cells per sample. Although not yet routinely performed the clinical demand for single-cell genotyping, as performed in our study, will likely increase, together with the use of NGS and targeted therapies, and the accordingly increased focus on clonal architecture and evolution of cancer.

In conclusion, we present an efficient workflow for the genetic analysis of individual cancer cells using the SCP for automated cell isolation. Given the flexibility of the SCP and its improved precision in cell deposition we were able to reliably use the instrument in combination with routine downstream genetic applications. In the future, we will combine the SCP with robotic liquid handling for further assay automation, corroborate our workflow for use with biopsies and explore the application of our workflow for NGS and the concurrent analyses of genetic and epigenetic aberrations.

## Supporting Information

S1 Fig Whole genome amplification and PCR on single Kasumi-1 cells. (PDF)

S2 Fig Whole genome amplification and PCR on single AML patient cells. (PDF)

S3 Fig Whole genome amplification and PCR on empty droplets. (PDF)

S4 Fig Allele peak plot of chromosome arm 17p in the AML patient. (PDF)

S1 Table Sequences of the primers used for the single-cell analyses. (PDF)
