# Supplementary material for: Molecular Genetic Characterization of Individual Cancer Cells Isolated via Single-Cell Printing
Source: PLoS One. 2016 Sep 22;11(9):e0163455. doi: 10.1371/journal.pone.0163455 (PMC5033393; doi:10.1371/journal.pone.0163455)
Supplement: S1 Fig — (PDF) [file pone.0163455.s001.pdf]

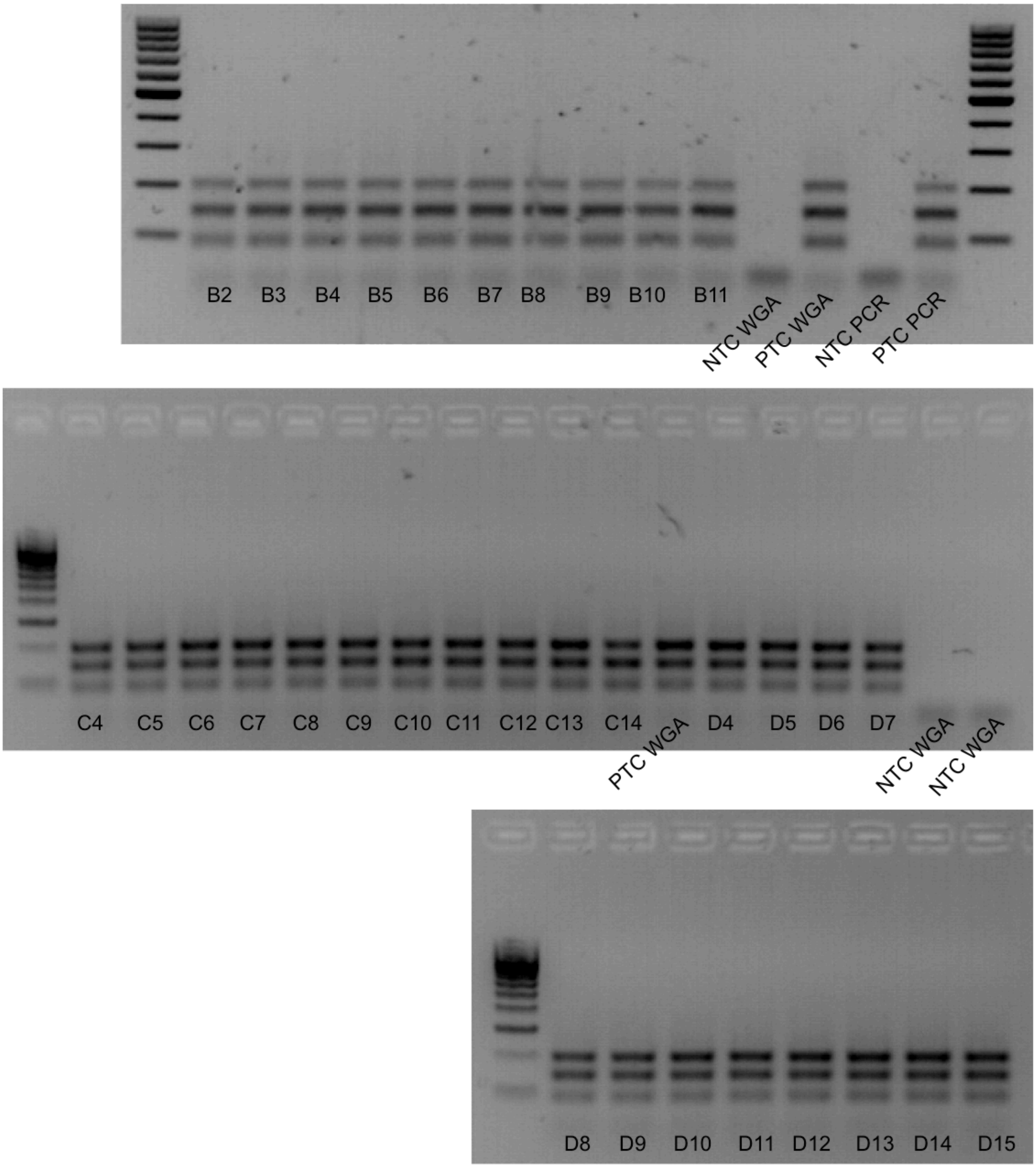

S1 Fig. Whole genome amplification and PCR on single Kasumi-1 cells

Multiplex PCR on *LINE1* retrotransposons after cell lysis and whole genome amplification (WGA) of single Kasumi-1 cells isolated by the SCP. The cell annotation corresponds to that in Fig. 5. For the NTC WGA, the WGA reaction was performed in a well of the 384-microwell plate in which no cell was deposited by the SCP. NTC, no-template control; PTC, positive control
