# Supplementary material for: Molecular Genetic Characterization of Individual Cancer Cells Isolated via Single-Cell Printing
Source: PLoS One. 2016 Sep 22;11(9):e0163455. doi: 10.1371/journal.pone.0163455 (PMC5033393; doi:10.1371/journal.pone.0163455)
Supplement: S2 Fig — (PDF) [file pone.0163455.s002.pdf]

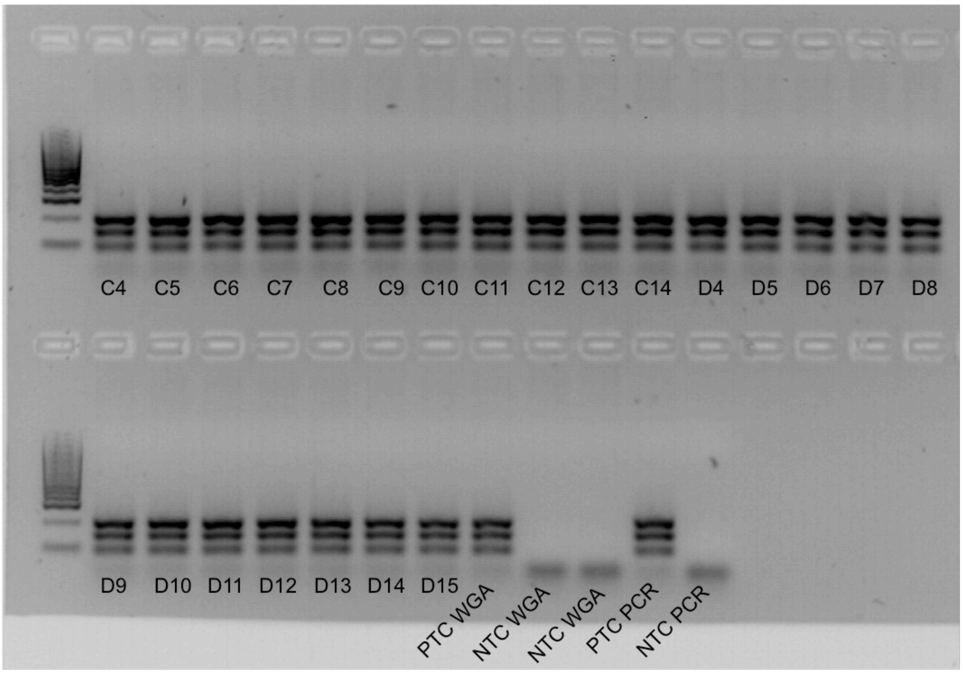

S2 Fig. Whole genome amplification and PCR on single AML patient cells

Multiplex PCR on *LINE1* retrotransposons after cell lysis and whole genome amplification (WGA) on single AML cells isolated by the SCP. The cell annotation corresponds to that in Figure 5. For the NTC WGA, the WGA reaction was performed in a well of the 384-microwell plate in which no cell was deposited by the SCP. NTC, no-template control; PTC, positive control
