# Supplementary material for: Molecular Genetic Characterization of Individual Cancer Cells Isolated via Single-Cell Printing
Source: PLoS One. 2016 Sep 22;11(9):e0163455. doi: 10.1371/journal.pone.0163455 (PMC5033393; doi:10.1371/journal.pone.0163455)
Supplement: S3 Fig — (PDF) [file pone.0163455.s003.pdf]

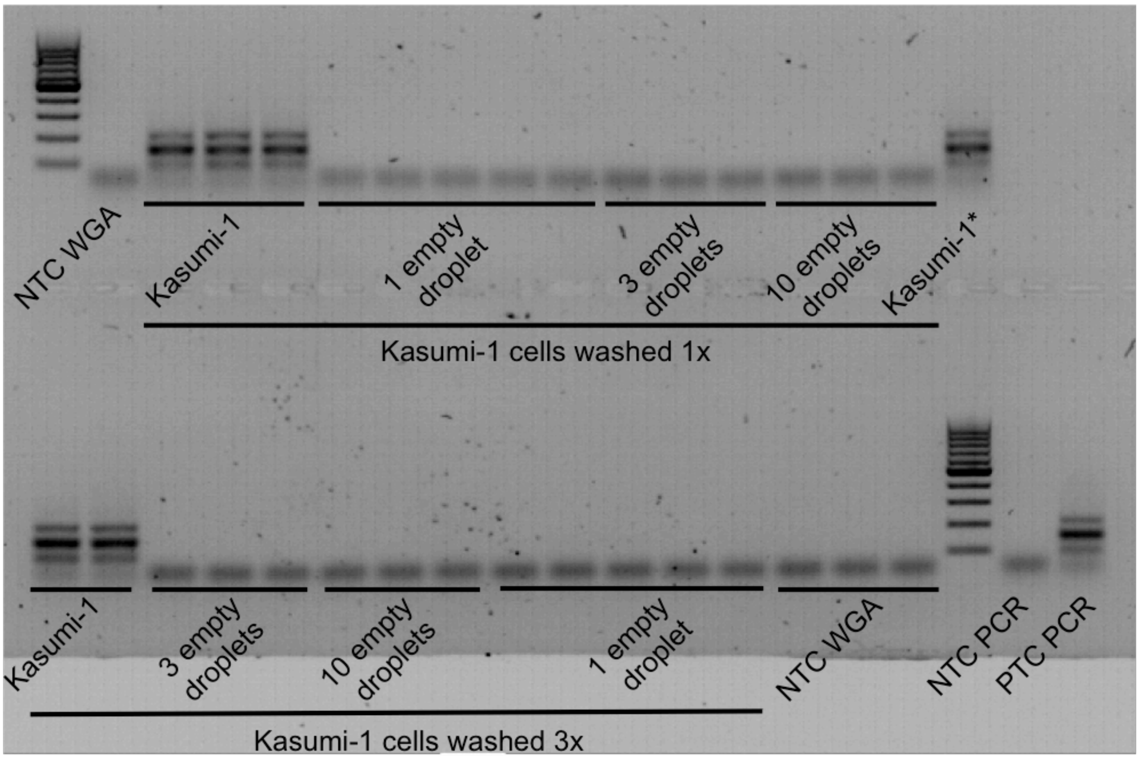

S3 Fig. Whole genome amplification and PCR on empty droplets

Multiplex PCR on *LINE1* retrotransposons after whole genome amplification (WGA) was performed on single Kasumi-1 cells and empty droplets. Kasumi-1 cells were either washed once or three times before they were filled into the Single-Cell Printer (SCP) cartridge; then, either Kasumi-1 cells or one, three or ten empty droplets were deposited into each well of a 384-microwell plate and subjected to cell lysis, WGA and *LINE1* PCR. * Kasumi-1 cells washed three times. NTC, no-template control; PTC, positive control
