# Supplementary material for: Molecular Genetic Characterization of Individual Cancer Cells Isolated via Single-Cell Printing
Source: PLoS One. 2016 Sep 22;11(9):e0163455. doi: 10.1371/journal.pone.0163455 (PMC5033393; doi:10.1371/journal.pone.0163455)
Supplement: S4 Fig — (PDF) [file pone.0163455.s004.pdf]

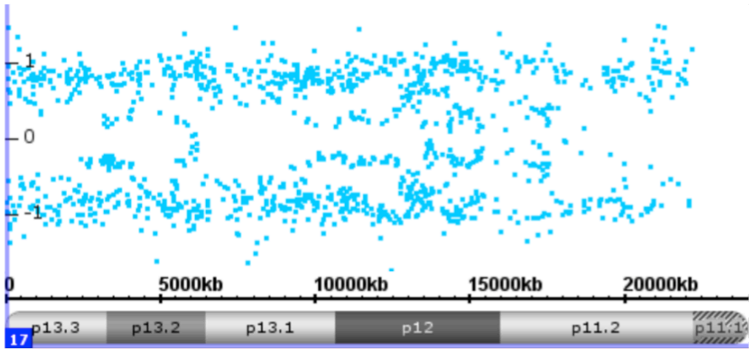

S4 Fig. Allele peak plot of chromosome arm 17p in the AML patient

Allele peak plot of chromosome arm 17p as assessed by CytoScan HD array in a peripheral blood sample of the patient AML. A homozygous AA genotype maps to approximately +1, a homozygous BB to approximately -1, a heterozygous AB to approximately 0 and a single A or B allele to 0.5 and -0.5, respectively. The allele peak plot indicates the loss of the chromosome 17p region in a subset of cells.
