# Supplementary material for: Molecular Genetic Characterization of Individual Cancer Cells Isolated via Single-Cell Printing
Source: PLoS One. 2016 Sep 22;11(9):e0163455. doi: 10.1371/journal.pone.0163455 (PMC5033393; doi:10.1371/journal.pone.0163455)
Supplement: S1 Table — (PDF) [file pone.0163455.s005.pdf]

S1 Table. Sequences of the primers used for the single-cell analyses.

KIT-F	TCATTCAAGGCGTACTTTTG
KIT-R	TCGAAAGTTGAAACTAAAAATCC
LINE1-F1	TGTAGGGTTTCTGCCGAGAG
LINE1-R1	GTTAAGGGCAGCCAGAGAGA
LINE1-F2	AACCCGACCTTTCTCTCTGG
LINE1-R2	GGCCAACGTTCAGATTCAGG
SLC34A2-F	CCTCACCTGTCCAACCTCTT
SLC34A2-R	GAACCAGCGATACTTGGCAG
TET2-F	ACCACTACCCCAACCAAAGT
TET2-R	TCATTGTCCCTGCAGTCTGT
TP53-F (Kasumi-1)	GCCACAGGTCTCCCCAAGGC
TP53-R (Kasumi-1)	TGGGGCACAGCAGGCCAGTG
TP53-F (Patient)	GGGACTGACTTTCTGCTCTT
TP53-R (Patient)	ATACGGCCAGGCATTGAAGT
rs1391438-F	CTAATGACTGGCTAAGCTCTTTT
rs1391438-R	TGAACTGTCCATGTCCCTTG
rs7655890-F	TGTGCAGTTGGTTTAATGGCA
rs7655890-R	TGCCATCTATGTTTTGGAGAAGT
